# Chlorido(η^4^-1,5-cyclo­octa­diene)[(penta­fluoro­eth­yl)diphenyl­phosphane]iridium(I)

**DOI:** 10.1107/S160053681005141X

**Published:** 2010-12-15

**Authors:** Michelle M. Choate, R. Gregory Peters, Russell G. Baughman

**Affiliations:** a Department of Chemistry, Wilkes University, Wilkes-Barre, PA 18766, USA; b Department of Chemistry, Truman State University, Kirksville, MO 63501-4221, USA

## Abstract

The title structure,[IrCl(C_8_H_12_)(C_14_H_10_F_5_P)], reveals that (C_2_F_5_)PPh_2_ (penta­fluoro­ethyl­diphenyl­phosphane or pfepp) disrupts the iridium dimer [(cod)IrCl]_2_ (cod = cyclo­octa-1,5-diene) by rupturing the bridging chloride ligands and binding in the open coordination site to form (cod)Ir(pfepp)Cl with the Ir^I^ atom in a distorted square-planar coordination environment. The structure deviates very little from the Ir^I^–triphenyl­phosphine analog, although a significantly (∼20σ) shorter Ir—P bond is noted for the title compound.

## Related literature

The structure of (cod)IrPPh_3_ was reported by Lebel & Ladjel (2008 ▶). The synthesis and crystal structure of pfepp has been reported by Palcic *et al.* (2004 ▶).
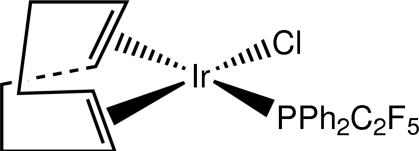


## Experimental

### 

#### Crystal data

[IrCl(C_8_H_12_)(C_14_H_10_F_5_P)]
*M* *_r_* = 640.02
Monoclinic, 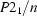

*a* = 10.5498 (5) Å
*b* = 14.9824 (7) Å
*c* = 13.9885 (7) Å
β = 94.579 (5)°
*V* = 2203.98 (18) Å^3^
*Z* = 4
Mo *K*α radiation
μ = 6.30 mm^−1^
*T* = 295 K
0.51 × 0.40 × 0.12 mm

#### Data collection

Bruker P4 diffractometer
Absorption correction: integration (*XSHELL*; Bruker, 1999 ▶) *T*_min_ = 0.155, *T*_max_ = 0.480
6244 measured reflections
5014 independent reflections
3583 reflections with *I* > 2σ(*I*)
*R*_int_ = 0.050
3 standard reflections every 100 reflections  intensity decay: 1.3%

#### Refinement

*R*[*F* ^2^ > 2σ(*F* ^2^)] = 0.042
*wR*(*F* ^2^) = 0.101
*S* = 1.03
5014 reflections
272 parameters
H-atom parameters constrained
Δρ_max_ = 1.08 e Å^−3^
Δρ_min_ = −1.31 e Å^−3^

### 

Data collection: *XSCANS* (Bruker, 1996 ▶); cell refinement: *XSCANS*; data reduction: *XSCANS*; program(s) used to solve structure: *SHELXS86* (Sheldrick, 2008 ▶); program(s) used to refine structure: *SHELXL97* (Sheldrick, 2008 ▶); molecular graphics: *SHELXTL/PC* (Sheldrick, 2008 ▶); software used to prepare material for publication: *SHELXTL/PC* and *SHELXL97*.

## Supplementary Material

Crystal structure: contains datablocks I, global. DOI: 10.1107/S160053681005141X/bq2260sup1.cif

Structure factors: contains datablocks I. DOI: 10.1107/S160053681005141X/bq2260Isup2.hkl

Additional supplementary materials:  crystallographic information; 3D view; checkCIF report

## Figures and Tables

**Table:** 

Ir1—C15	2.100 (8)
Ir1—C16	2.125 (9)
Ir1—C19	2.209 (9)
Ir1—C20	2.235 (8)
Ir1—P1	2.2705 (17)
Ir1—Cl1	2.352 (2)

**Table:** 

P1—Ir1—Cl1	89.89 (7)
C7—P1—C1	100.8 (3)
C7—P1—C13	101.3 (3)
C1—P1—C13	102.7 (3)
C7—P1—Ir1	115.1 (2)
C1—P1—Ir1	121.4 (2)
C13—P1—Ir1	112.8 (3)

**Fig. 1.**
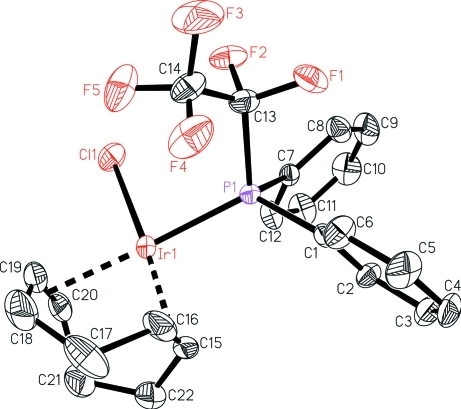
The asymmetric unit of (I) showing the labeling of the non-H atoms. Displacement ellipsoids are drawn at the 30% probability levels; H atoms have been omitted for clarity.

## supplementary crystallographic information

### Comment

The mean deviation of the Ir1/Cl1/P1/*Cg*1/*Cg*2 plane (*Cg*1 = centroid of C15/C16; *Cg*2 = centroid of C19/C20), is 0.064Å, indicating that Ir1 is in a square planar environment. The *pfepp* ligand and the chloride occupy the sites *trans* to the two centroids of the cycloocta-1,5-diene (cod) ligand. The Ir1—*Cg*1 and Ir1—*Cg*2 centroid distances are 2.00 (2) and 2.12 (2) Å, respectively, a difference of 0.12 Å (= 6σ). As seen in Fig. 1, the chloride ligand is *trans* to the shorter distance, possibly due to the high electronegativity of the chloride, which attracts the olefinic electrons and results in a shorter centroid distance. The Ir-centroids bond angle of 86.0 (3)° is compressed from the ideal 90°, while the P1—Ir1—Cl1 bond angle [89.89 (7)°] is essentially ideal.

The title compound is structurally very similar to the PPh_3_ analog (Lebel & Ladjel, 2008) with the only noticeable difference coming in the Ir1—P1 bond length, which is shorter by 0.040 Å (~20σ) in the title complex. The three torsion angles Cl1—Ir1—P1—C(1, 7, or 13) of the title compound have values within 5° of the counterpart angles in the PPh_3_ structure, indicating minimal structural effects on the ligand upon substitution of a phenyl group with a —C_2_F_5_.

### Experimental

The title complex was prepared by the addition of an excess of pentafluoroethyldiphenylphosphane (C_2_F_5_)PPh_2_ (*pfepp*) to a heptane solution of [(cod)IrCl]_2_ under nitrogen. This solution was refluxed for two hours and allowed to cool to room temperature; the title compound was isolated by filtration in 79% yield. Single crystals were grown by the slow evaporation of a diethyl ether solution at room temperature. In addition to the single-crystal structure determination of this complex, multinuclear NMR spectra (^1^H, ^19^F, and ^31^P), IR and elemental analysis were consistent with the X-ray structure.

### Refinement

Approximate positions of the majority of the H's were first obtained from a difference map, then placed into ideal positions and refined as a rotational group. Bond lengths were constrained at 0.93 Å (AFIX 43) for aromatic and ethylenic C—H's; and 0.97 Å (AFIX 23) for methylene C—H's. *U*_iso_(H) were fixed at 1.2 *U*_eq_(parent) for all H's.

In the final stages of refinement, 5 reflections with very small or negative *F*_o_'s were deemed to be in high disagreement with their *F*_c_'s and were eliminated from final refinement.

### Crystal data

**Table:**

[IrCl(C_8_H_12_)(C_14_H_10_F_5_P)]	*F*(000) = 1232
*M**_r_* = 640.02	*D*_x_ = 1.929 Mg m^−^^3^
Monoclinic, *P*2_1_/*n*	Mo *K*α radiation, λ = 0.71073 Å
Hall symbol: -P 2yn	Cell parameters from 100 reflections
*a* = 10.5498 (5) Å	θ = 11.0–18.7°
*b* = 14.9824 (7) Å	µ = 6.30 mm^−^^1^
*c* = 13.9885 (7) Å	*T* = 295 K
β = 94.579 (5)°	Parallelepiped, orange
*V* = 2203.98 (18) Å^3^	0.51 × 0.40 × 0.12 mm
*Z* = 4	

### Data collection

**Table:**

Bruker P4 diffractometer	3583 reflections with *I* > 2σ(*I*)
Radiation source: normal-focus sealed tube	*R*_int_ = 0.050
graphite	θ_max_ = 27.5°, θ_min_ = 2.0°
θ/2θ scans	*h* = −1→13
Absorption correction: integration (*XSHELL*; Bruker, 1999)	*k* = −1→19
*T*_min_ = 0.155, *T*_max_ = 0.480	*l* = −18→18
6244 measured reflections	3 standard reflections every 100 reflections
5014 independent reflections	intensity decay: 1.3%

### Refinement

**Table:**

Refinement on *F*^2^	Secondary atom site location: difference Fourier map
Least-squares matrix: full	Hydrogen site location: difference Fourier map
*R*[*F*^2^ > 2σ(*F*^2^)] = 0.042	H-atom parameters constrained
*wR*(*F*^2^) = 0.101	*w* = 1/[σ^2^(*F*_o_^2^) + (0.041*P*)^2^ + 4.9533*P*] where *P* = (*F*_o_^2^ + 2*F*_c_^2^)/3
*S* = 1.03	(Δ/σ)_max_ = 0.001
5014 reflections	Δρ_max_ = 1.08 e Å^−^^3^
272 parameters	Δρ_min_ = −1.31 e Å^−^^3^
0 restraints	Extinction correction: *SHELXL97* (Sheldrick, 2008), *F*_c_*=k*F*_c_[1+0.001x*F*_c_^2^λ^3^/sin(2θ)]^-1/4^
Primary atom site location: structure-invariant direct methods	Extinction coefficient: 0.00082 (14)

### Special details

**Table:**

Geometry. All s.u.'s (except the s.u. in the dihedral angle between two l.s. planes) are estimated using the full covariance matrix. The cell s.u.'s are taken into account individually in the estimation of s.u.'s in distances, angles and torsion angles; correlations between s.u.'s in cell parameters are only used when they are defined by crystal symmetry. An approximate (isotropic) treatment of cell s.u.'s is used for estimating s.u.'s involving l.s. planes.
Refinement. Refinement of *F*^2^ against ALL reflections. The weighted *R*-factor, *wR*, and goodness of fit, *S*, are based on *F*^2^, conventional *R*-factors, *R*, are based on *F*, with *F* set to zero for negative *F*^2^. The threshold expression of *F*^2^ > 2σ(*F*^2^) is used only for calculating *R*-factors(gt) *etc*. and is not relevant to the choice of reflections for refinement. *R*-factors based on *F*^2^ are statistically about twice as large as those based on *F*, and *R*-factors based on ALL data will be even larger.

### Fractional atomic coordinates and isotropic or equivalent isotropic displacement parameters (Å^2^)

**Table:**

	*x*	*y*	*z*	*U*_iso_*/*U*_eq_	
Ir1	0.09989 (2)	0.327209 (17)	0.78744 (2)	0.03420 (11)	
Cl1	0.2697 (2)	0.23154 (16)	0.7593 (2)	0.0625 (6)	
P1	−0.00925 (16)	0.20976 (11)	0.84243 (12)	0.0283 (4)	
F1	−0.0049 (5)	0.1050 (3)	1.0012 (4)	0.0620 (13)	
F2	0.1605 (5)	0.0925 (3)	0.9191 (4)	0.0652 (14)	
F3	0.1897 (8)	0.1652 (5)	1.1018 (5)	0.109 (3)	
F4	0.0877 (6)	0.2798 (4)	1.0506 (5)	0.090 (2)	
F5	0.2575 (6)	0.2431 (5)	0.9899 (5)	0.094 (2)	
C1	−0.1697 (7)	0.2233 (4)	0.8808 (5)	0.0337 (15)	
C2	−0.2707 (7)	0.2092 (5)	0.8127 (6)	0.0416 (17)	
H2	−0.2548	0.1924	0.7508	0.050*	
C3	−0.3940 (8)	0.2197 (6)	0.8357 (8)	0.056 (2)	
H3	−0.4604	0.2100	0.7892	0.067*	
C4	−0.4206 (8)	0.2445 (6)	0.9266 (8)	0.064 (3)	
H4	−0.5040	0.2502	0.9426	0.077*	
C5	−0.3202 (9)	0.2605 (7)	0.9933 (7)	0.067 (3)	
H5	−0.3363	0.2783	1.0548	0.080*	
C6	−0.1962 (8)	0.2503 (6)	0.9710 (6)	0.051 (2)	
H6	−0.1300	0.2621	1.0171	0.061*	
C7	−0.0332 (6)	0.1169 (4)	0.7591 (5)	0.0326 (15)	
C8	−0.0646 (8)	0.0314 (5)	0.7865 (6)	0.0479 (19)	
H8	−0.0629	0.0155	0.8509	0.057*	
C9	−0.0992 (9)	−0.0307 (6)	0.7137 (8)	0.066 (3)	
H9	−0.1204	−0.0887	0.7299	0.079*	
C10	−0.1015 (10)	−0.0072 (7)	0.6199 (8)	0.068 (3)	
H10	−0.1249	−0.0494	0.5731	0.081*	
C11	−0.0716 (10)	0.0753 (6)	0.5928 (7)	0.061 (2)	
H11	−0.0743	0.0899	0.5281	0.073*	
C12	−0.0361 (8)	0.1391 (5)	0.6618 (6)	0.0476 (19)	
H12	−0.0140	0.1963	0.6434	0.057*	
C13	0.0785 (8)	0.1526 (5)	0.9502 (6)	0.0469 (19)	
C14	0.1525 (9)	0.2123 (7)	1.0235 (7)	0.062 (2)	
C15	−0.0647 (8)	0.4023 (5)	0.7499 (7)	0.052 (2)	
H15	−0.1095	0.3489	0.7435	0.063*	
C16	−0.0193 (10)	0.4263 (5)	0.8418 (8)	0.066 (3)	
H16	−0.0298	0.3871	0.8921	0.079*	
C17	0.0482 (14)	0.5150 (7)	0.8636 (9)	0.094 (4)	
H17A	0.0147	0.5594	0.8179	0.113*	
H17B	0.0299	0.5348	0.9271	0.113*	
C18	0.1904 (13)	0.5092 (7)	0.8593 (10)	0.099 (4)	
H18A	0.2223	0.5673	0.8423	0.119*	
H18B	0.2289	0.4942	0.9226	0.119*	
C19	0.2299 (10)	0.4429 (7)	0.7903 (8)	0.070 (3)	
H19	0.2940	0.4040	0.8130	0.084*	
C20	0.1848 (9)	0.4311 (6)	0.6972 (7)	0.063 (3)	
H20	0.2205	0.3860	0.6624	0.076*	
C21	0.0793 (10)	0.4874 (7)	0.6471 (8)	0.076 (3)	
H21A	0.0863	0.5477	0.6721	0.091*	
H21B	0.0925	0.4901	0.5793	0.091*	
C22	−0.0485 (10)	0.4553 (7)	0.6571 (9)	0.080 (3)	
H22A	−0.0741	0.4174	0.6027	0.096*	
H22B	−0.1057	0.5061	0.6550	0.096*	

### Atomic displacement parameters (Å^2^)

**Table:**

	*U*^11^	*U*^22^	*U*^33^	*U*^12^	*U*^13^	*U*^23^
Ir1	0.02825 (15)	0.03151 (14)	0.04259 (17)	−0.00469 (13)	0.00129 (10)	0.00204 (14)
Cl1	0.0344 (11)	0.0660 (13)	0.0886 (17)	0.0093 (9)	0.0145 (11)	0.0040 (12)
P1	0.0271 (8)	0.0267 (8)	0.0309 (9)	0.0012 (7)	0.0017 (7)	0.0006 (7)
F1	0.078 (3)	0.053 (3)	0.055 (3)	0.005 (3)	0.010 (3)	0.020 (2)
F2	0.064 (3)	0.061 (3)	0.069 (3)	0.034 (3)	−0.001 (3)	0.003 (3)
F3	0.126 (6)	0.126 (6)	0.066 (4)	0.013 (5)	−0.045 (4)	0.018 (4)
F4	0.077 (4)	0.096 (4)	0.093 (5)	0.013 (4)	−0.023 (4)	−0.046 (4)
F5	0.061 (4)	0.139 (6)	0.080 (4)	−0.025 (4)	−0.008 (3)	−0.020 (4)
C1	0.035 (4)	0.028 (3)	0.039 (4)	0.001 (3)	0.012 (3)	0.004 (3)
C2	0.030 (4)	0.049 (4)	0.046 (4)	0.006 (3)	0.006 (3)	0.000 (3)
C3	0.035 (4)	0.049 (5)	0.085 (7)	0.002 (4)	0.004 (4)	−0.010 (5)
C4	0.032 (5)	0.058 (5)	0.105 (8)	0.000 (4)	0.026 (5)	0.003 (5)
C5	0.055 (6)	0.091 (7)	0.059 (6)	0.020 (5)	0.035 (5)	−0.001 (5)
C6	0.044 (5)	0.068 (5)	0.043 (5)	0.008 (4)	0.012 (4)	−0.009 (4)
C7	0.029 (3)	0.029 (3)	0.041 (4)	0.001 (3)	0.007 (3)	−0.005 (3)
C8	0.052 (5)	0.036 (4)	0.057 (5)	−0.003 (3)	0.011 (4)	−0.004 (4)
C9	0.069 (6)	0.034 (4)	0.097 (8)	−0.011 (4)	0.015 (5)	−0.023 (5)
C10	0.078 (7)	0.060 (6)	0.067 (7)	−0.005 (5)	0.011 (5)	−0.029 (5)
C11	0.081 (7)	0.059 (5)	0.045 (5)	−0.011 (5)	0.016 (5)	−0.020 (4)
C12	0.056 (5)	0.044 (4)	0.044 (5)	−0.013 (4)	0.013 (4)	−0.004 (4)
C13	0.045 (4)	0.050 (5)	0.046 (4)	0.013 (4)	0.004 (4)	0.007 (3)
C14	0.051 (6)	0.084 (7)	0.048 (5)	0.005 (5)	−0.018 (4)	−0.001 (5)
C15	0.037 (4)	0.039 (4)	0.083 (7)	0.008 (3)	0.014 (4)	0.014 (4)
C16	0.079 (7)	0.030 (4)	0.091 (8)	0.008 (4)	0.028 (6)	−0.004 (4)
C17	0.163 (14)	0.042 (5)	0.079 (8)	−0.008 (7)	0.030 (8)	−0.017 (5)
C18	0.116 (11)	0.054 (6)	0.121 (11)	−0.031 (7)	−0.034 (9)	−0.011 (7)
C19	0.061 (6)	0.057 (6)	0.088 (8)	−0.026 (5)	−0.018 (5)	0.023 (5)
C20	0.057 (6)	0.057 (5)	0.076 (7)	−0.020 (4)	0.005 (5)	0.029 (5)
C21	0.085 (8)	0.078 (7)	0.064 (6)	−0.014 (6)	0.003 (6)	0.031 (6)
C22	0.068 (7)	0.068 (7)	0.098 (9)	−0.002 (5)	−0.020 (6)	0.033 (6)

### Geometric parameters (Å, °)

**Table:**

Ir1—C15	2.100 (8)	C9—C10	1.356 (14)
Ir1—C16	2.125 (9)	C9—H9	0.9301
Ir1—C19	2.209 (9)	C10—C11	1.338 (13)
Ir1—C20	2.235 (8)	C10—H10	0.9298
Ir1—P1	2.2705 (17)	C11—C12	1.388 (11)
Ir1—Cl1	2.352 (2)	C11—H11	0.9297
P1—C7	1.819 (7)	C12—H12	0.9301
P1—C1	1.827 (7)	C13—C14	1.527 (12)
P1—C13	1.908 (8)	C15—C16	1.383 (14)
F1—C13	1.376 (10)	C15—C22	1.542 (13)
F2—C13	1.344 (9)	C15—H15	0.9300
F3—C14	1.335 (11)	C16—C17	1.526 (14)
F4—C14	1.294 (11)	C16—H16	0.9300
F5—C14	1.321 (12)	C17—C18	1.509 (18)
C1—C6	1.375 (11)	C17—H17A	0.9700
C1—C2	1.388 (10)	C17—H17B	0.9700
C2—C3	1.373 (11)	C18—C19	1.469 (17)
C2—H2	0.9299	C18—H18A	0.9700
C3—C4	1.374 (14)	C18—H18B	0.9700
C3—H3	0.9295	C19—C20	1.361 (14)
C4—C5	1.376 (15)	C19—H19	0.9297
C4—H4	0.9298	C20—C21	1.522 (14)
C5—C6	1.377 (11)	C20—H20	0.9301
C5—H5	0.9299	C21—C22	1.449 (14)
C6—H6	0.9295	C21—H21A	0.9702
C7—C8	1.385 (10)	C21—H21B	0.9698
C7—C12	1.399 (11)	C22—H22A	0.9699
C8—C9	1.406 (12)	C22—H22B	0.9700
C8—H8	0.9298		
			
C15—Ir1—C16	38.2 (4)	F1—C13—C14	105.6 (7)
C15—Ir1—C19	94.8 (4)	F2—C13—P1	109.2 (5)
C16—Ir1—C19	80.2 (4)	F1—C13—P1	110.6 (5)
C15—Ir1—C20	81.2 (3)	C14—C13—P1	117.2 (6)
C16—Ir1—C20	89.5 (4)	F4—C14—F5	108.0 (9)
C19—Ir1—C20	35.7 (4)	F4—C14—F3	107.4 (9)
C15—Ir1—P1	93.9 (2)	F5—C14—F3	106.0 (8)
C16—Ir1—P1	95.2 (3)	F4—C14—C13	113.6 (7)
C19—Ir1—P1	158.9 (3)	F5—C14—C13	111.4 (8)
C20—Ir1—P1	165.4 (3)	F3—C14—C13	110.0 (8)
C15—Ir1—Cl1	155.5 (3)	C16—C15—C22	126.5 (9)
C16—Ir1—Cl1	165.0 (3)	C16—C15—Ir1	71.9 (5)
C19—Ir1—Cl1	90.1 (3)	C22—C15—Ir1	110.0 (6)
C20—Ir1—Cl1	89.1 (3)	C16—C15—H15	116.7
P1—Ir1—Cl1	89.89 (7)	C22—C15—H15	116.8
C7—P1—C1	100.8 (3)	Ir1—C15—H15	88.2
C7—P1—C13	101.3 (3)	C15—C16—C17	122.3 (9)
C1—P1—C13	102.7 (3)	C15—C16—Ir1	69.9 (5)
C7—P1—Ir1	115.1 (2)	C17—C16—Ir1	113.6 (8)
C1—P1—Ir1	121.4 (2)	C15—C16—H16	119.0
C13—P1—Ir1	112.8 (3)	C17—C16—H16	118.8
C6—C1—C2	118.2 (7)	Ir1—C16—H16	86.5
C6—C1—P1	124.3 (6)	C18—C17—C16	113.0 (9)
C2—C1—P1	117.4 (5)	C18—C17—H17A	109.0
C3—C2—C1	120.8 (8)	C16—C17—H17A	109.0
C3—C2—H2	119.6	C18—C17—H17B	109.0
C1—C2—H2	119.6	C16—C17—H17B	109.0
C2—C3—C4	121.0 (9)	H17A—C17—H17B	107.8
C2—C3—H3	119.5	C19—C18—C17	113.6 (9)
C4—C3—H3	119.5	C19—C18—H18A	108.8
C3—C4—C5	118.2 (8)	C17—C18—H18A	108.8
C3—C4—H4	121.0	C19—C18—H18B	108.8
C5—C4—H4	120.8	C17—C18—H18B	108.8
C4—C5—C6	121.3 (9)	H18A—C18—H18B	107.7
C4—C5—H5	119.4	C20—C19—C18	128.4 (11)
C6—C5—H5	119.3	C20—C19—Ir1	73.2 (5)
C1—C6—C5	120.5 (8)	C18—C19—Ir1	109.5 (8)
C1—C6—H6	119.7	C20—C19—H19	115.8
C5—C6—H6	119.8	C18—C19—H19	115.8
C8—C7—C12	120.1 (7)	Ir1—C19—H19	87.0
C8—C7—P1	123.7 (6)	C19—C20—C21	123.7 (11)
C12—C7—P1	115.6 (5)	C19—C20—Ir1	71.1 (5)
C7—C8—C9	117.7 (8)	C21—C20—Ir1	109.6 (6)
C7—C8—H8	121.1	C19—C20—H20	118.0
C9—C8—H8	121.2	C21—C20—H20	118.3
C10—C9—C8	120.9 (9)	Ir1—C20—H20	89.3
C10—C9—H9	119.5	C22—C21—C20	115.0 (8)
C8—C9—H9	119.6	C22—C21—H21A	108.6
C11—C10—C9	121.8 (9)	C20—C21—H21A	108.6
C11—C10—H10	119.0	C22—C21—H21B	108.4
C9—C10—H10	119.2	C20—C21—H21B	108.5
C10—C11—C12	119.8 (9)	H21A—C21—H21B	107.5
C10—C11—H11	120.2	C21—C22—C15	114.8 (8)
C12—C11—H11	120.0	C21—C22—H22A	108.7
C11—C12—C7	119.8 (8)	C15—C22—H22A	108.4
C11—C12—H12	120.1	C21—C22—H22B	108.6
C7—C12—H12	120.1	C15—C22—H22B	108.6
F2—C13—F1	106.0 (6)	H22A—C22—H22B	107.6
F2—C13—C14	107.6 (7)		
			
C15—Ir1—P1—C7	99.6 (4)	P1—C13—C14—F5	−73.4 (9)
C16—Ir1—P1—C7	137.9 (4)	F2—C13—C14—F3	−67.3 (10)
C19—Ir1—P1—C7	−146.1 (9)	F1—C13—C14—F3	45.6 (10)
C20—Ir1—P1—C7	29.9 (11)	P1—C13—C14—F3	169.3 (7)
Cl1—Ir1—P1—C7	−56.2 (3)	C19—Ir1—C15—C16	−67.4 (6)
C15—Ir1—P1—C1	−22.3 (4)	C20—Ir1—C15—C16	−100.5 (6)
C16—Ir1—P1—C1	16.0 (4)	P1—Ir1—C15—C16	93.4 (5)
C19—Ir1—P1—C1	92.0 (9)	Cl1—Ir1—C15—C16	−168.2 (5)
C20—Ir1—P1—C1	−92.0 (11)	C16—Ir1—C15—C22	123.1 (9)
Cl1—Ir1—P1—C1	−178.1 (3)	C19—Ir1—C15—C22	55.7 (8)
C15—Ir1—P1—C13	−144.8 (4)	C20—Ir1—C15—C22	22.7 (7)
C16—Ir1—P1—C13	−106.4 (4)	P1—Ir1—C15—C22	−143.5 (7)
C19—Ir1—P1—C13	−30.4 (9)	Cl1—Ir1—C15—C22	−45.1 (10)
C20—Ir1—P1—C13	145.6 (11)	C22—C15—C16—C17	4.0 (15)
Cl1—Ir1—P1—C13	59.4 (3)	Ir1—C15—C16—C17	105.8 (10)
C7—P1—C1—C6	146.8 (7)	C22—C15—C16—Ir1	−101.8 (9)
C13—P1—C1—C6	42.4 (7)	C19—Ir1—C16—C15	110.9 (6)
Ir1—P1—C1—C6	−84.8 (7)	C20—Ir1—C16—C15	76.3 (6)
C7—P1—C1—C2	−36.4 (6)	P1—Ir1—C16—C15	−89.8 (5)
C13—P1—C1—C2	−140.8 (6)	Cl1—Ir1—C16—C15	160.9 (8)
Ir1—P1—C1—C2	92.1 (6)	C15—Ir1—C16—C17	−117.4 (10)
C6—C1—C2—C3	−1.7 (12)	C19—Ir1—C16—C17	−6.5 (8)
P1—C1—C2—C3	−178.8 (6)	C20—Ir1—C16—C17	−41.1 (8)
C1—C2—C3—C4	0.1 (13)	P1—Ir1—C16—C17	152.8 (8)
C2—C3—C4—C5	1.4 (14)	Cl1—Ir1—C16—C17	43.5 (17)
C3—C4—C5—C6	−1.4 (16)	C15—C16—C17—C18	−90.8 (14)
C2—C1—C6—C5	1.8 (13)	Ir1—C16—C17—C18	−10.5 (14)
P1—C1—C6—C5	178.7 (7)	C16—C17—C18—C19	30.8 (16)
C4—C5—C6—C1	−0.3 (15)	C17—C18—C19—C20	48.4 (16)
C1—P1—C7—C8	−66.2 (7)	C17—C18—C19—Ir1	−35.2 (13)
C13—P1—C7—C8	39.3 (7)	C15—Ir1—C19—C20	−67.6 (7)
Ir1—P1—C7—C8	161.4 (6)	C16—Ir1—C19—C20	−103.0 (7)
C1—P1—C7—C12	105.0 (6)	P1—Ir1—C19—C20	178.3 (6)
C13—P1—C7—C12	−149.5 (6)	Cl1—Ir1—C19—C20	88.4 (7)
Ir1—P1—C7—C12	−27.4 (6)	C15—Ir1—C19—C18	58.0 (8)
C12—C7—C8—C9	−0.3 (12)	C16—Ir1—C19—C18	22.6 (8)
P1—C7—C8—C9	170.5 (7)	C20—Ir1—C19—C18	125.6 (11)
C7—C8—C9—C10	0.1 (14)	P1—Ir1—C19—C18	−56.2 (13)
C8—C9—C10—C11	−0.2 (16)	Cl1—Ir1—C19—C18	−146.0 (8)
C9—C10—C11—C12	0.5 (16)	C18—C19—C20—C21	−0.5 (15)
C10—C11—C12—C7	−0.7 (14)	Ir1—C19—C20—C21	101.4 (8)
C8—C7—C12—C11	0.6 (12)	C18—C19—C20—Ir1	−101.9 (11)
P1—C7—C12—C11	−171.0 (7)	C15—Ir1—C20—C19	111.2 (7)
C7—P1—C13—F2	38.6 (6)	C16—Ir1—C20—C19	73.8 (7)
C1—P1—C13—F2	142.6 (6)	P1—Ir1—C20—C19	−177.5 (9)
Ir1—P1—C13—F2	−85.0 (6)	Cl1—Ir1—C20—C19	−91.3 (7)
C7—P1—C13—F1	−77.7 (6)	C15—Ir1—C20—C21	−8.9 (8)
C1—P1—C13—F1	26.3 (6)	C16—Ir1—C20—C21	−46.3 (8)
Ir1—P1—C13—F1	158.7 (4)	C19—Ir1—C20—C21	−120.1 (11)
C7—P1—C13—C14	161.2 (7)	P1—Ir1—C20—C21	62.4 (16)
C1—P1—C13—C14	−94.8 (7)	Cl1—Ir1—C20—C21	148.6 (8)
Ir1—P1—C13—C14	37.5 (7)	C19—C20—C21—C22	−87.9 (13)
F2—C13—C14—F4	172.2 (8)	Ir1—C20—C21—C22	−8.0 (13)
F1—C13—C14—F4	−74.9 (10)	C20—C21—C22—C15	28.3 (15)
P1—C13—C14—F4	48.8 (11)	C16—C15—C22—C21	46.7 (15)
F2—C13—C14—F5	50.0 (10)	Ir1—C15—C22—C21	−35.1 (12)
F1—C13—C14—F5	162.8 (7)		
